# Mortality trends and years of potential life lost due to suicide in adolescents

**DOI:** 10.11606/s1518-8787.2024058005564

**Published:** 2024-07-10

**Authors:** Beatriz Catarina dos Santos de Oliveira, Ruth Ellery Lima Flores, Amanda Cristina de Souza Andrade, Roberta Mendes Abreu Silva, Katiene Rodrigues Menezes de Azevedo, Vanessa Moraes Bezerra

**Affiliations:** I Universidade Federal da Bahia Instituto Multidisciplinar em Saúde Vitória da Conquista BA Brasil Universidade Federal da Bahia. Instituto Multidisciplinar em Saúde. Vitória da Conquista, BA, Brasil; II Universidade Federal de Mato Grosso Instituto de Saúde Coletiva Cuiabá MT Brasil Universidade Federal de Mato Grosso. Instituto de Saúde Coletiva. Cuiabá, MT, Brasil; III Secretaria de Vigilância em Saúde e Ambiente Brasília DF Brasil Secretaria de Vigilância em Saúde e Ambiente. Brasília, DF, Brasil

**Keywords:** Mortality, Suicide, Adolescent, Time Series Studies, Years of Potential Life Lost

## Abstract

**OBJECTIVE:**

To assess the trend in mortality rates and years of potential life lost (YPLL) due to suicide among adolescents in Northeast Brazil.

**METHODS:**

This is an ecological time series study, with secondary data from 2011 to 2020 from the Mortality Information System for adolescents aged 10 to 19 years in the Northeast region of Brazil. Groups of causes from the 10th Revision of the International Classification of Diseases were included: X60-X84 (intentionally self-inflicted injuries), Y10-Y19 (poisoning of undetermined intent), and Y87 (sequelae of intentional self-harm). Mortality coefficients and frequency distribution by sociodemographic variables, place of occurrence, and method of suicide were estimates. YPLL were estimated by gender and age. Joinpoint regression analysis was used, and the annual percentage change (APC) was determined with 95% confidence intervals.

**RESULTS:**

A total of 2,410 deaths were recorded, with a predominance of adolescents aged between 15 and 19, males, of mixed-race, low schooling, and home was the main place of occurrence. The trend in the death rate was increasing in the Northeast (APC: 3.6%; p = 0.001), in girls aged 10 to 14 (APC: 8.7%; p = 0.003), in boys aged 15 to 19 (APC: 4.6%; p = 0.002) and in Bahia (APC: 8.1%; p = 0.012). Hanging/strangulation was the main method adopted by both sexes. The YPLL due to suicide were 11,110 in 2011 and 14,960 in 2020.

**CONCLUSION:**

The precociousness of suicide committed by girls and the increase in mortality among older adolescents are noteworthy, and specific preventive measures need to be adopted for these groups in order to reduce this preventable cause of death.

## INTRODUCTION

Suicide is self-injurious behavior aimed at death^1^ and can be understood as the last event in a cascade that characterizes suicidal behavior. In Brazil, in 2019, the suicide rate was 6.6/100,000 inhabitants, of which 23.3% of cases occurred among adolescents^2^. From 2010 to 2019, there was an 81% increase in suicide mortality rates within this group^2^. This increase may be associated with generational particularities, especially generation Z (“born digital,” after 1995), which is more vulnerable to emotional, socioeconomic, and cultural issues^1,2^.

Adolescence is a phase marked by greater psychological, sexual, and social development. The adolescent population has its own characteristics which make them more vulnerable to suicide, such as emotional immaturity, progressive family distancing, loneliness, and difficulty in joining groups^1,3^. In addition, adolescents are more likely to develop mental health problems, especially if they have suffered some kind of violence, and may respond less to protective factors, which increases the risk of suicidal behavior^4^.

The phenomenon of suicide has consequences that go beyond the family and affect society as a whole^5^. As the adolescent population is composed of young people, their productive lives may be interrupted by a potentially preventable death. It is estimated that each case of suicide affects at least six other people and can have an impact on their emotional and social spheres^5^. For this reason, this topic is considered a public health matter^6^.

Due to the wide cultural and economic diversity among Brazilian regions, the risk factors for suicide are not evenly distributed. In 2019, the Northeast region had the lowest suicide rates among adolescents^2^. However, studies reveal an increasing temporal trend of deaths from this cause in the region^1,7^. This situation may be linked to the fact that the Northeast has high social vulnerability compared to other regions of Brazil^8^.

Among the indicators used to show the profile of deaths in Brazil is the years of potential life lost (YPLL), a rate that indicates how many years an individual has lost due to premature death. In the context of suicide in adolescents, this allows quantifying the impact of early and preventable mortality and also to presume social and emotional repercussions caused by it^9^.

In view of this, this study assessed the trend in the suicide mortality rate among adolescents in the Northeast region of Brazil and the YPLL due to this cause. The results obtained may contribute to the development of preventive actions and a reduction in mortality in the country, in line with the WHO’s global targets of reducing suicide deaths by a third by 2030^6^.

## METHODS

This is an ecological time series study of suicide mortality in the adolescent population (10 to 19 years old) living in the Northeast region of Brazil from 2011 to 2020.

Secondary data from the Mortality Information System (SIM) and resident population estimates calculated by the Brazilian Institute of Geography and Statistics (IBGE) were used, both available on the platform of the Information Technology Department of the Unified Health System (Datasus).

To define the suicide outcome, we considered deaths whose underlying causes were classified according to the tenth revision of the International Classification of Diseases (ICD-10), including the groups of causes X60 to X69 (intentional autointoxication), X70 to X84 (intentionally self-inflicted injuries), Y10 to Y19 (exogenous intoxication of undetermined intent), and Y87 (sequelae of intentional self-harm). The last two were included in the analysis considering that the literature describes the change in classification of deaths due to possible faults in the coding of the cause^10^.

The absolute and relative frequencies of suicide mortality were described according to sociodemographic variables: gender, age group (10 to 14 years; 15 to 19 years), race/color (White, Black, Yellow, mixed and Indigenous), years of schooling (none, 1 to 3 years, 4 to 7 years, 8 to 11 years, and 12 or more years) and place of suicide (hospital, other health facility, home, public road, and others).

The methods used to commit suicide were presented by ICD-10 category groupings: X60-X64 (intentional self-intoxication with drugs), X65-X69 (intentional self-intoxication with chemicals), X70 (hanging/strangulation), X72-X75 (firearms and explosives), X76 (smoke, fire and flames), X78 e X79 (sharp and blunt objects), X80 and X81 (jumping from a high place and standing in front of a moving object), X71, X82-X84 (other self-harm—drowning by submersion, motor vehicle impact and other means), Y11-Y19 (poisoning of undetermined intent) e Y87 (sequelae of intentional self-harm).

Suicide mortality rates were stratified by sex, age group, and federative units in the Northeast region. To calculate the rates, the absolute values of codes X60-X84, Y10-Y19, and Y87 were added together. The number of deaths for each variable wasconsidered the numerator and the respective estimate of the adolescent population the denominator, multiplied by 100,000 inhabitants.

Under-reporting of deaths was rectified by applying the mortality rate correction factors produced of Szwarcwald et al.^11^, who carried out an active search in 129 municipalities of the Northeast and of the Legal Amazon and estimated the coverage of vital information systems in this region.^11^

Data on YPLL were estimated according to age group and gender. The index values per individual were obtained from the difference between the upper age limit, set at 70 years old^12^, and the mean age for each age group, in which the midpoint was equal to*: (youngest age in the age group in years + oldest age + 1)/2*^13^. The individual YPLL were then multiplied by the corresponding number of deaths to generate the total values by age group and sex.

The rates were calculated using the Excel program, version 2019. For the trend analysis, the dependent variable was the mortality rate and the independent variable was the years of the historical series. Joinpoint regression analysis was used, and the annual percentage change (APC) and its respective 95% confidence intervals (95%CI) were calculated, with a significance level of 5%, for sex, age group, Northeast region, as well as for its federative units. The assumptions of serial autocorrelation, normality and homoscedasticity were checked using the Durbin-Watson, Shapiro-Wilk, and Breusch-Pagan tests, respectively. When serial autocorrelation was verified, the correlated error model was used. These statistical analyses were carried out using the Joinpoint Regression Program software, version 4.9.1.0.

## RESULTS

From 2011 to 2020, 2,410 deaths by suicide were recorded among adolescents in the Northeast region of Brazil.

The 15–19 age group accounted for the highest percentage for both sexes (85%), with males predominating. The most affected individuals were those classified as mixed-race (75%), followed by Whites (12.4%). As for schooling, the highest frequencies were found among those with up to 7 years of schooling and, in 22.2% of cases, schooling was not reported. Regarding the place of occurrence, the highest percentages were found at home (57.1%) and in hospital (20.9%). The percentage of deaths occurring in hospitals and other health facilities was higher for females. Deaths on public roads and in other places were higher among males (Table 1).

**Table 1:** Proportional distribution of deaths by suicide among adolescents living in the Northeast region, according to sociodemographic variables and place of occurrence, from 2011 to 2020.

Characteristic	Boys		Girls		Total	
		
	n	%	n	%	n	%
Age (years)						
10–14	199	12.4	162	20.1	361	15.0
15–19	1,405	87.6	644	79.9	2,049	85.0
Race/color						
White	175	10.9	125	15.5	300	12.4
Black	79	4.9	48	6.0	127	5.3
Yellow	2	0.1	1	0.1	3	0.1
Mixed	1,232	76.8	575	71.3	1,807	75.0
Indigenous	10	0.6	6	0.7	16	0.7
Unknown	106	6.6	51	6.3	157	6.5
Years of study						
None	27	1.7	8	1.0	35	1.5
1–3	209	13.0	75	9.3	284	11.8
4–7	574	35.8	276	34.2	850	35.3
8–11	385	24.0	265	32.9	650	27.0
≥ 12	33	2.1	22	2.7	55	2.3
Unknown	376	23.4	160	19.9	536	22.2
Place of occurrence						
Hospital	255	15.9	249	30.9	504	20.9
Other health facility	22	1.4	24	3.0	46	1.9
Home	960	59.9	416	51.6	1,376	57.1
Public road	115	7.2	42	5.2	157	6.5
Other	245	15.3	73	9.1	318	13.2
Unknown	7	0.4	2	0.2	9	0.4

**Figure 1:**
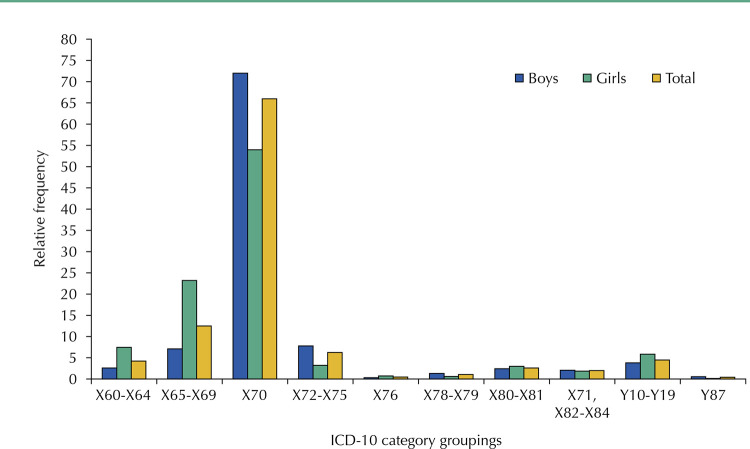
Distribution of methods used to commit suicide by adolescents, according to ICD-10 category groupings, Northeast Region, 2011–2020.

X60-X64: intentional self-poisoning by drugs; X65-X69: intentional self-intoxication with chemicals; X70: hanging/strangulation; X72-X75: firearms and explosives; X76: smoke, fire and flames; X78 and X79: sharp and blunt objects; X80-81: jumping from a high place and standing in front of a moving object; X71, X82-X84: other self-harm; Y10-Y19: poisoning of undetermined intent; Y87: sequelae of intentional self-harm.

As for the methods used for suicide, hanging/strangulation was the main cause for males (72%), followed by the use of firearms and explosives (7.8%). Among girls, deaths by hanging/strangulation accounted for 54%, followed by 23.2% cases of intentional self-intoxication by chemical substances (Figure 1).

In relation to suicide mortality rates, the highest rates were observed among males (15 to 19 years old). There was also an increase in the rate for the total population, from 1.96/100,000 inhabitants, in 2011, to 2.75, in 2020. The only period in which there was a reduction in mortality was between 2017 and 2018. Among the states of the Northeast region, the lowest rate was observed in Bahia and the highest in Piauí (Table 2).

**Table 2:** Corrected mortality rates (100,000 inhabitants) due to suicide among adolescents, by sex, age group, and federal units in the Northeast region of Brazil, from 2011 to 2020.

Characteristic	2011	2012	2013	2014	2015	2016	2017	2018	2019	2020
Gender/age group										
Boys 10–14 years	1.03	0.86	0.82	1.03	0.78	0.92	1.06	0.49	0.86	0.88
Boys 15–19 years	4.47	5.77	6.06	5.87	6.31	6.46	7.48	6.08	7.58	7.52
Girls 10–14 years	0.54	0.44	0.76	0.53	0.63	0.68	1.01	0.75	1.14	0.97
Girls 15–19 years	3.09	2.79	2.56	2.84	2.88	3.18	3.49	3.08	2.76	3.11
Northeast region	2.26	2.43	2.51	2.54	2.62	2.80	3.26	2.61	3.12	3.16
Federative unit										
Alagoas	1.34	2.85	2.51	2.84	1.34	2.52	1.69	2.04	1.89	3.48
Bahia	1.18	1.45	1.29	1.56	1.49	2.04	2.34	1.30	2.06	3.13
Ceará	3.16	3.72	4.16	3.90	3.72	3.11	4.15	3.83	4.55	3.80
Maranhão	2.52	2.22	2.78	2.95	4.16	3.29	3.74	2.70	3.17	2.34
Paraíba	2.45	2.62	2.45	1.31	4.08	2.12	3.42	3.42	3.26	1.96
Pernambuco	2.08	2.40	2.00	1.68	2.07	2.72	3.05	2.02	2.37	2.53
Piauí	3.91	3.70	3.89	6.19	2.70	4.43	6.35	5.23	6.66	4.59
Rio Grande do Norte	3.37	1.58	1.78	2.17	1.58	3.56	2.58	3.18	3.41	4.25
Sergipe	2.90	2.62	4.17	2.60	4.15	3.38	3.91	3.15	3.45	4.30

**Figure 2:**
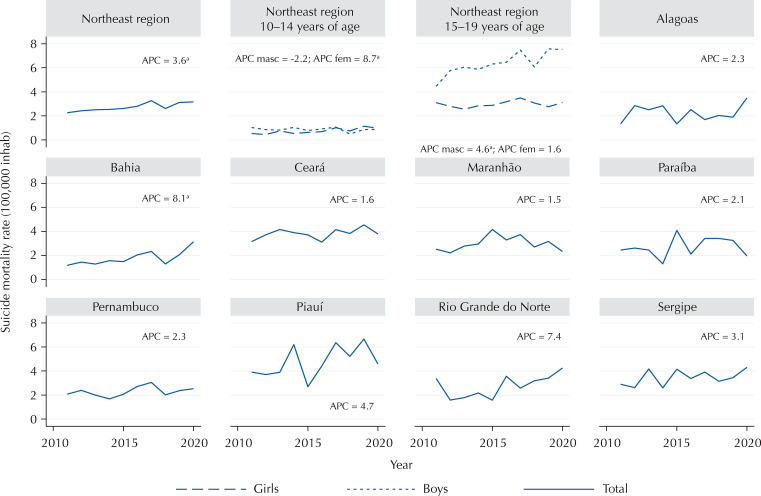
Temporal trend of corrected suicide mortality rates among adolescents in the Northeast region, according to sex, age group, and federative units, from 2011 to 2020.

^a^p < 0,05

There was an upward trend in the suicide mortality rate in the Northeast. Among adolescents aged 10 to 14, there was a stationary trend for males and an increasing trend for females. In the 15–19 age group, the opposite was observed: an increasing trend for boys and a stationary trend for girls. Among the states of the Northeast region, most showed a stationary trend, with the exception of Bahia, which showed an increasing trend (Figure 2).

APC: annual percentage change.

Suicide deaths among adolescents represented a loss of 11,110 YPLL in 2011 and 14,960 in 2020. The highest figures were among individuals aged 15 to 19 years, especially males. In the total population, there was an increase in the YPLL rate from 2011 to 2020. In females, the increase was from 26.8 to 48.3 among younger adolescents, while in males the rate decreased over the period studied. Among older adolescents, both sexes presented an increase in the YPLL rate (Table 3).

**Table 3:** Years of potential life lost (YPLL) due to suicide among adolescents in the Northeast region of Brazil, according to sex and age group, from 2011 to 2020.

Age group	Gender	Deaths		YPLL^a^	Mean age point	Total YPLL		YPLL/100.000	
			
		2011	2020	2011/2020		2011	2020	2011	2020
10–14 years		**36**	**39**	**57.5**	**12.5**	**2,070.0**	**2,242.5**	**39.5**	**46.0**
	Boys	24	19	57.5	12.5	1,380.0	1,092.5	51.7	43.9
	Girls	12	20	57.5	12.5	690.0	1,150.0	26.8	48.3
15–19 years		**166**	**233**	**52.5**	**17.5**	**8,715.0**	**12,232.5**	**173.0**	**243.8**
	Boys	99	166	52.5	17.5	5,197.5	8,715.0	204.1	343.5
	Girls	67	67	52.5	17.5	3,517.5	3,517.5	141.3	141.,8
Total		**202**	**272**	**55.0**	**15.0**	**11,110.0**	**14,960.0**	**108.1**	**151.3**

^a^ YPLL by individual.

## DISCUSSION

From 2011 to 2020, there was an increasing trend in the suicide mortality rate among adolescents in the Northeast region of Brazil. The highest rates were observed in the 15–19 age group, rising in males and stable in females. Most deaths occurred among adolescents of mixed-race and low schooling. Home was the main place of occurrence, and hanging/strangulation the main used method. There was an increase in YPLL in the period studied.

The increasing trend in mortality rates observed may be associated with the changes instituted in the care network during the study period, such as the focus on hospitalization and the dismantling of community-based health services^14^. Among those affected by the new National Mental Health Policy are the *Centro de Atenção Psicossocial* (CAPS – Psychosocial Care Centers), which have seen a sharp reduction in the number of new units set up^14^, implying a lack of psychological and psychiatric care for adolescents. Considering that mental disorders are risk factors for suicide^5,15^, reduced access to the Psychosocial Care Network can increase the risk for this population.

We must also consider the influence of the use of social media by adolescents of the current generation. Evidence shows that excessive screen time is related to mental illnesses^16^. Prolonged exposure to social media has been associated with poorer sleep quality, cyberbullying, and greater internalization of problems, resulting in augmented risk of suicidal behaviour^16^. Evidence also indicates that the increase in these rates may be related to widespread news coverage of celebrity suicide, a phenomenon known as the contagion effect or Werther effect, in which individuals feel encouraged to reproduce this behavior^17^. Together with the characteristics of the current generation, such as low resilience and immediacy, this may have influenced the increase in suicide rates^2^. On the other hand, social networks also have a positive potential with the support networks that are formed among internet users, highlighting the need to create and strengthen virtual prevention strategies to reach this vulnerable population^16^.

The predominance of deaths by suicide among adolescents of mixed-race has already been pointed out in a study conducted in Bahia^18^. It is known that most of the Northeastern population declare themselves to as mixed^19^, and this group is among those classified as being more socially vulnerable (lower incomes, informal jobs, etc.)^20^ Suicide rates among adolescents are positively associated with socioeconomic inequalities, reinforcing the suspicion that the development characteristics of this region may influence the risk of death by suicide^21^.

In the same vein, higher percentages of suicides were observed among male individuals with less schooling. Low academic performance and poor school adherence have also been identified as risk factors for suicide^15^. Many adolescents are unable to combine study and work, as well as having less encouragement from their parents, who mostly have low educational level^20^.

The home was the main place where suicides were committed. In addition to highlighting the importance of family support in preventing self-harm, this data reinforces the protective or risk role that the family can play in suicidal behavior^22^, since family problems increase the risk of suicide^23^. It is also assumed that the higher proportion at home is related to both the ease of obtaining the tools used to commit suicide, and the speed with which death is achieved according to the method^1,18^.

Hospitals were identified as the second most common place for suicide, and were more prevalent among female adolescents. Their choice of methods is generally less lethal, indicating a lower desire for death and the need for help, since there are more reports of attempted suicide and hospitalizations for autointoxication among them^3^. However, if the attempt to reverse the method is unsuccessful, hospital mortality records are high^1,24^. Boys, on the other hand, generally opt for more violent methods with a high risk of death^25^. This may explain the higher occurrence of deaths among this group at home, on public roads and in other places that do not provide immediate medical assistance.

Analysis of the means used strengthens this understanding. Although hanging/strangulation was the main form of suicide for both sexes, as well as the use of firearms, these percentages were higher for boys. Explanations for this behavior include cultural issues that associate masculinity with independence and making more definitive decisions, as well as avoiding the social repercussions of using a method that enables them to survive and face the facts that led them to attempt suicide^15^. On the other hand, girls died more from autointoxication by drugs and other chemical substances, as well as poisoning of undetermined intent, which may be associated with a greater occurrence of depression and a possible desire to survive, since they are more likely to ask for and accept help to deal with their problems^15^.

The mortality rates found in this study reinforce this hypothesis, as the highest figures predominated among boys in both age groups. Other studies conducted in the Northeast and other Brazilian regions have also found a higher incidence of suicide mortality among boys^7,21,24^. Regarding age groups, the predominance of deaths occurring between 15 and 19 years of age in both sexes may reflect constant exposure to risk factors specific to this age, such as difficulties inherent in the transition phase to adulthood, greater stress and anxiety regarding professional and academic future, excessive use of psychoactive substances and problems in family and social relationships^1,7,15^. In addition, evidence points to an association between suicide and adolescents who are not linked to a formal education, employment or training system^15^.

The temporal trend of the suicide mortality rate among adolescents in the Northeast was increasing over the period studied, which was similar to that observed in other studies^1,7,26^. In the 10 to 14 age group, there was an increasing trend for girls and stability for boys, unlike another study carried out in the region between 2001 and 2015, in which boys showed an upward trend whereas girls showed stability^7^. The change in mortality trends among girls aged 10 to 14, compared to the previous study, may be due to their greater exposure to peer conflicts, bullying and experiences of emotional, sexual, or physical abuse, which have already been identified as risk factors for suicidal behavior, especially in young girls^15^. However, regarding the 15–19 age group, the current study agrees with the evidence of a previous one, which showed an increase in the mortality trend for boys and stability for girls^7^. This may be due to the fact that boys are more inclined to hide their weaknesses and adopt a more aggressive approach to choosing lethal methods of suicide than girls^25,27^.

Regarding the states in the Northeast region, although Bahia had one of the lowest suicide mortality rates, it was the only state that showed an increasing trend, with an annual percentage variation greater than that observed in the region. This trend has already been highlighted by other studies, which analyzed the periods from 1997 to 2016^1^ and 2001 to 2015^7^ and may be related to sociodemographic aspects, as the state of Bahia still has high income inequality, especially in terms of race/color, in which the average household income of self-declared Blacks and mixed-race is lower than that of Whites. In addition, according to 2019 data, the illiteracy rate among 15-year-old Black adolescents was higher than that of Whites in Bahia^28^ and it is known that schooling and other factors, can determine employment and income opportunities^20^.

The results of this study revealed that there were differences in YPLL due to suicide according to age and gender. Although the youngest age group showed an increase in the 2020 rate compared to 2011, it was observed that this increase was attributable only to an increase in the female rate, while the male rate showed a decrease. This draws attention to an increasingly early search for a definitive solution to internal and external conflicts experienced by female adolescents and the factors that could be related to this precocity, such as psychological and/or sexual violence, stressful events, excessive use of social media, and lack of family support^3,5,16,26^

In relation to older adolescents, an increase in the YPLL rate was observed in both sexes, and was more significant in males. This can be attributed not only to the issues that are generally associated with this group, such as greater abstention from school, exposure to alcohol, and unemployment^15^, but also to an aggravation by the context of the COVID-19 pandemic, which began in 2020, when there was an increase in the occurrence of suicides in the Northeast region, although Brazil showed a general pattern of decline^29^. The pandemic, in addition to the emotional losses, has reduced the possibilities of employment and continuing studies, especially for those from public schools and without adequate resources to maintain remote education. Data shows that the reduction in occupation affected many young people in 2020 and, for this population, recovery after crises takes even longer^8^. In addition, the pandemic context has favored mental illnesses by producing a scenario of fear of contagion and the need for social isolation, which has increased stress and anxiety levels in both healthy individuals and those previously diagnosed with mental disorders^30^. The sum of these factors may have increased the likelihood of suicide.

The findings of this study reinforce the relevance of death by suicide among adolescents as a public health problem, while at the same time helping with the epidemiological understanding of this phenomenon. Among the limitations are the restrictions of the databases which only provide information by sex, while the literature points to the significant influence of gender characteristics on the risk of suicide. In addition, there is still a large number of ignored fields for the education variable and the underreporting of suicide cases, either due to the difficulty of determining the intentionality of the death or as only the type of injury is considered, without taking into account the context in which it was produced. Although other ICD-10 categories have been included in an attempt to minimize the impact of underreporting, it should be noted that other classification errors may have occurred. The consequent poor completion of death certificates has repercussions that limit the implementation and improvement of specific prevention policies for vulnerable populations.

The increasing trend in mortality among adolescents in the Northeast region and in the state of Bahia reinforces the weight of sociodemographic characteristics in determining the vulnerability that confers a greater risk of suicide in these places. The predominance of mortality among adolescents aged 15 to 19, especially males, has repercussions on the increase in YPLL in a population of productive age. The increasing trend in the mortality rate among girls aged 10 to 14 draws attention to the early existence of factors that cause mental illnesses and the growing search for drastic measures to resolve conflicts. From this perspective, the different susceptibility profiles among adolescents point to the need of adopting specific approaches and forms of prevention for the factors that most predispose each group to suicide, as well as the relevance of exploiting the protective potential of the internet, by strengthening online support communities, search engines and offering virtual help. Moreover, there is a need to broaden the view of risk factors, which go beyond emotional aspects and are influenced by a comprehensive panorama, such as the socio-cultural context, access to the care network, school, employment, and income opportunities.

The results of this study made it possible to identify the profile of the most affected individuals, the most common forms of execution and how differences between sexes can influence the occurrence of suicide. There is a need for specific interventions to contribute towards more effective public policies to promote health and prevent suicide among adolescents.
