# Supplementary material for: Mortality trends and years of potential life lost due to suicide in adolescents
Source: Rev Saude Publica. 2024 Jul 10;58:30. doi: 10.11606/s1518-8787.2024058005564 (PMC11319033; doi:10.11606/s1518-8787.2024058005564)
Supplement: Translation

# Translation: Tendência da mortalidade e anos potenciais de vida perdidos por suicídio de adolescentes

**Authors:** Beatriz Catarina dos Santos de Oliveira, Ruth Ellery Lima Flores, Amanda Cristina de Souza Andrade, Roberta Mendes Abreu Silva, Katiene Rodrigues Menezes de Azevedo, Vanessa Moraes Bezerra

**Affiliations:** I Brasil Universidade Federal da Bahia. Instituto Multidisciplinar em Saúde. Vitória da Conquista, BA, Brasil; II Brasil Universidade Federal de Mato Grosso. Instituto de Saúde Coletiva. Cuiabá, MT, Brasil; III Brasil Secretaria de Vigilância em Saúde e Ambiente. Brasília, DF, Brasil

**Keywords:** Mortalidade, Suicídio, Adolescente, Estudos de Séries Temporais, Anos Potenciais de Vida Perdidos

## Abstract

**OBJETIVO:**

Avaliar a tendência das taxas de mortalidade e os anos potenciais de vida perdidos (APVP) por suicídio entre adolescentes do Nordeste brasileiro.

**MÉTODOS:**

Estudo ecológico de séries temporais, com dados secundários de 2011 a 2020 do Sistema de Informações sobre Mortalidade, de adolescentes entre 10 e 19 anos no Nordeste do Brasil. Foram incluídos os grupos de causas da 10ª Revisão da Classificação Internacional de Doenças: X60-X84 (lesões autoprovocadas intencionalmente), Y10-Y19 (intoxicação de intenção indeterminada) e Y87 (sequela de lesões autoprovocadas intencionalmente). Calculou-se os coeficientes de mortalidade e a distribuição de frequências por variáveis sociodemográficas, local de ocorrência e método de suicídio adotado. Os APVP foram estimados por sexo e idade. Foi utilizada a análise de regressão por *Joinpoint* e determinada a variação percentual anual (APC) com intervalos de confiança de 95%.

**RESULTADOS:**

Registrou-se 2.410 óbitos, com predomínio de adolescentes entre 15 e 19 anos, sexo masculino, raça/cor parda, baixa escolaridade e, como principal local de ocorrência, o domicílio. A tendência da taxa de óbitos foi crescente no Nordeste (APC: 3,6%; p = 0,001),em meninas de 10 a 14 anos (APC: 8,7%; p = 0,003), em meninos de 15 a 19 anos (APC: 4,6%;p = 0,002) e na Bahia (APC: 8,1%; p = 0,012). Enforcamento/estrangulamento foi o principal método adotado em ambos os sexos. Os APVP por suicídio foram de 11.110 anos em 2011 e de 14.960 em 2020.

**CONCLUSÕES:**

Chama a atenção a precocidade do suicídio cometido pelas meninas e o aumento da mortalidade entre os adolescentes mais velhos, sendo necessária a adoção de medidas preventivas específicas para esses grupos, a fim de reduzir esta causa evitável de morte.

## INTRODUÇÃO

O suicídio é um comportamento autolesivo com intenção de morte^1^ e pode ser entendido como o último evento de uma cascata que caracteriza o comportamento suicida. No Brasil, em 2019, a taxa de suicídio foi de 6,6/100.000 habitantes, sendo que 23,3% dos casos de lesões autoprovocadas ocorreu entre os adolescentes^2^. No período de 2010 a 2019 houve um aumento de 81% nas taxas de mortalidade por suicídio nesse grupo^2^. Este aumento pode estar associado a particularidades geracionais, sobretudo da geração Z (“natos digitais”, nascidos após 1995), que está mais vulnerável a questões emocionais, socioeconômicas e culturais^1,2^.

A adolescência é uma fase marcada por maior desenvolvimento psicológico, sexual e de relações sociais. A população adolescente carrega características próprias, que a confere maior vulnerabilidade ao suicídio, tais como imaturidade emocional, distanciamento familiar progressivo, solidão e dificuldade de inserção em grupos^1,3^. Além disso, os adolescentes têm maior probabilidade de desenvolver algum problema de saúde mental, principalmente se sofreram algum tipo de violência, podendo responder menos aos fatores de proteção, o que aumenta o risco para o comportamento suicida^4^.

O fenômeno do suicídio traz consequências que ultrapassam o âmbito familiar e alcançam toda a sociedade^5^. Na população adolescente, por ser formada por pessoas jovens, a vida produtiva é interrompida por um óbito potencialmente evitável. Estima-se que cada caso de suicídio afete ao menos outras seis pessoas, podendo impactar suas esferas emocionais e sociais^5^, dessa forma, o tópico é considerado como um problema de saúde pública^6^.

Devido à ampla diversidade cultural e econômica entre as regiões brasileiras, os fatores de risco para o suicídio não se distribuem de forma homogênea. Em 2019, a região Nordeste apresentou as menores taxas de suicídio entre adolescentes^2^, contudo, estudos apontam uma tendência temporal crescente de óbitos por esta causa na região^1,7^. Situação que pode estar atrelada ao fato de o Nordeste possuir alta vulnerabilidade social em relação às demais regiões do país^8^.

Entre os indicadores utilizados para mostrar o perfil de óbitos no Brasil, tem-se os anos potenciais de vida perdidos (APVP), taxa que representa quantos anos um indivíduo deixou de viver por morte prematura. No contexto do suicídio em adolescentes, este indicador permite quantificar o impacto da mortalidade precoce e prevenível e, consequentemente, presumir as repercussões sociais e emocionais provocadas por ela^9^.

Diante disso, este estudo objetivou avaliar a tendência da taxa de mortalidade por suicídio entre adolescentes do Nordeste brasileiro e os anos potenciais de vida perdidos por essa causa. Assim, poderá contribuir para elaboração de ações preventivas e diminuição da mortalidade no país, em consonância com as metas globais da OMS de reduzir em um terço os óbitos por suicídio até 2030^6^.

## MÉTODOS

Trata-se de um estudo ecológico de séries temporais sobre a mortalidade por suicídio na população de adolescentes (10 a 19 anos) residentes na região Nordeste do Brasil, no período entre 2011 e 2020.

Foram utilizados dados secundários do Sistema de Informações sobre Mortalidade (SIM) e estimativas de população residente calculadas pelo Instituto Brasileiro de Geografia e Estatística (IBGE), ambos disponíveis na plataforma do Departamento de Informática do Sistema Único de Saúde (Datasus).

Para a definição do desfecho suicídio, foram considerados os óbitos cujas causas básicas foram classificadas de acordo com a décima revisão da Classificação Internacional de Doenças (CID-10), incluindo os grupos de causas X60 a X69 (autointoxicação intencional), X70 a X84 (lesões autoprovocadas intencionalmente), Y10 a Y19 (intoxicação exógena de intenção indeterminada) e Y87 (sequela de lesões autoprovocadas intencionalmente), os dois últimos foram incluídos na análise considerando-se que a literatura descreve a mudança de classificação dos óbitos devido a possíveis falhas na codificação da causa^10^.

As frequências absolutas e relativas da mortalidade por suicídio foram descritas segundo variáveis sociodemográficas: sexo, faixa etária (10 a 14 anos; 15 a 19 anos), raça/cor (branca, preta, amarela, parda e indígena), anos de estudo (nenhum, 1 a 3 anos, 4 a 7 anos, 8 a 11 anos e 12 ou mais anos) e local de ocorrência do suicídio (hospital, outro estabelecimento de saúde, domicílio, via pública e outros).

Os métodos utilizados para consumação do suicídio foram apresentados por agrupamentos de categorias da CID-10: X60-X64 (autointoxicação por fármacos), X65-X69 (autointoxicação por substâncias químicas), X70 (enforcamento/estrangulamento), X72-X75 (arma de fogo e explosivos), X76 (fumaça, fogo e chamas), X78 e X79 (objetos cortantes e contundentes), X80 e X81 (precipitação de lugar elevado e permanência diante de objeto em movimento), X71, X82-X84 (outras lesões autoprovocadas – afogamento por submersão, impacto de veículo a motor e outros meios), Y11-Y19 (envenenamento de intenção não determinada) e Y87 (sequela de lesão autoprovocada).

As taxas de mortalidade por suicídio foram estratificadas por sexo, faixa etária e Unidades Federativas da região Nordeste. Para os cálculos das taxas, realizou-se a soma dos valores absolutos dos códigos X60-X84, Y10-Y19 e Y87. O número de óbitos para cada variável foi considerado o numerador e a respectiva estimativa da população de adolescentes o denominador, multiplicado por 100.000 habitantes.

A correção de sub-registro de óbitos foi realizada pela aplicação dos fatores de correção das taxas de mortalidade produzidos por Szwarcwald et al.^11^, que realizou uma busca ativa em 129 municípios do Nordeste e da Amazônia Legal e estimou as coberturas dos sistemas de informações vitais nesta região.^11^

Os dados referentes aos APVP foram estimados segundo faixa etária e sexo. Os valores do índice, por indivíduo, foram obtidos pela diferença entre o limite superior de idade, fixado em 70 anos^12^, e o ponto médio de idade para cada faixa etária, em que o ponto médio foi igual a: *(idade mais jovem da faixa etária em anos + idade mais avançada + 1)/2*^13^. Os APVP individuais foram multiplicados pelo número de óbitos correspondentes para gerar os valores totais por faixa etária e sexo.

Os cálculos das taxas foram realizados no programa Excel versão 2019. Para a análise de tendência, foi considerada como variável dependente a taxa de mortalidade e variável independente os anos da série histórica. Foi utilizada a análise de regressão por *Joinpoint* e calculada a variação percentual anual (APC – *Annual Percent Change*) e seus respectivos intervalos de confiança de 95% (IC95%), com nível de significância de 5%, para sexo e faixa etária, Região Nordeste, bem como para suas Unidades Federativas. Foram verificadas as suposições de autocorrelação serial, normalidade e homoscedasticidade pelos testes de Durbin-Watson, Shapiro-Wilk e Breusch-Pagan, respectivamente. Quando verificada a autocorrelação serial, foi utilizado o modelo de erros correlacionados. Estas análises estatísticas foram realizadas com o *software Joinpoint Regression Program,* versão 4.9.1.0.

## RESULTADOS

No período de 2011 a 2020 foram registrados 2.410 óbitos por suicídio entre adolescentes da região Nordeste do Brasil.

A faixa etária de 15 a 19 anos representou o maior percentual para ambos os sexos (85%), e observou-se predomínio no sexo masculino. Os indivíduos mais acometidos foram os classificados como pardos (75%), seguidos dos brancos (12,4%). Quanto à escolaridade, as maiores frequências foram observadas entre aqueles com até 7 anos de estudos e, em 22,2% dos casos, a escolaridade não foi informada. Em relação ao local de ocorrência, observou-se maiores percentuais no domicílio (57,1%) e no hospital (20,9%). O percentual de óbitos ocorridos nos hospitais e em outros estabelecimentos de saúde foi maior para o sexo feminino. Já os óbitos ocorridos em via pública e em outros locais foram maiores entre os meninos (Tabela 1).

**Tabela 1:** Distribuição proporcional dos óbitos por suicídio ocorridos entre adolescentes residentes na região Nordeste, segundo variáveis sociodemográficas e local de ocorrência, no período de 2011 a 2020.

Variáveis	Masculino		Feminino		Total	
		
	n	%	n	%	n	%
Idade (anos)						
10–14	199	12,4	162	20,1	361	15,0
15–19	1405	87,6	644	79,9	2.049	85,0
Raça/cor						
Branca	175	10,9	125	15,5	300	12,4
Preta	79	4,9	48	6,0	127	5,3
Amarela	2	0,1	1	0,1	3	0,1
Parda	1.232	76,8	575	71,3	1807	75,0
Indígena	10	0,6	6	0,7	16	0,7
Ignorado	106	6,6	51	6,3	157	6,5
Anos de estudos						
Nenhum	27	1,7	8	1,0	35	1,5
1–3	209	13,0	75	9,3	284	11,8
4–7	574	35,8	276	34,2	850	35,3
8–11	385	24,0	265	32,9	650	27,0
≥ 12	33	2,1	22	2,7	55	2,3
Ignorado	376	23,4	160	19,9	536	22,2
Local de ocorrência						
Hospital	255	15,9	249	30,9	504	20,9
Outro estabelecimento de @@saúde	22	1,4	24	3,0	46	1,9
Domicílio	960	59,9	416	51,6	1376	57,1
Via pública	115	7,2	42	5,2	157	6,5
Outros	245	15,3	73	9,1	318	13,2
Ignorado	7	0,4	2	0,2	9	0,4

Quanto aos métodos utilizados para o suicídio, para o sexo masculino, o enforcamento/estrangulamento foi a principal causa (72%), seguido de uso de arma de fogo e explosivos (7,8%). Entre as meninas, os óbitos por enforcamento/estrangulamento representaram 54%, seguidos de 23,2% casos de autointoxicação intencional por substâncias químicas (Figura 1).

**Figura 1:** [Distribuição dos métodos utilizados para cometer suicídio por adolescentes, segundo agrupamentos das categorias da CID-10, Região Nordeste, 2011-2020.].

X60-X64: autointoxicação intencional por fármacos; X65-X69: autointoxicação intencional por substâncias químicas; X70: enforcamento/estrangulamento; X72-X75: arma de fogo e explosivos; X76: fumaça, fogo e chamas; X78 e X79: objetos cortantes e contundentes; X80-81: precipitação de lugar elevado e permanência diante de objeto em movimento; X71, X82-X84: outras lesões autoprovocadas; Y10-Y19: envenenamento de intenção não determinada; Y87: sequela de lesão autoprovocada.

Em relação às taxas de mortalidade por suicídio, as maiores foram observadas no sexo masculino (15 a 19 anos). Para a população total, foi observado um aumento da taxa, passando de 1,96/100.000 habitantes em 2011 para 2,75 em 2020. O único período que correspondeu a uma redução da mortalidade foi entre 2017 e 2018. Entre os estados da região Nordeste, a menor taxa foi observada na Bahia e a maior no Piauí (Tabela 2).

**Tabela 2:** Taxas de mortalidade (100.000 habitantes) por suicídio em adolescentes corrigidas, segundo sexo, faixa etária e unidades federativas da região Nordeste do Brasil, de 2011 a 2020.

Variáveis	2011	2012	2013	2014	2015	2016	2017	2018	2019	2020
Sexo/faixa etária										
Masculino 10–14 anos	1,03	0,86	0,82	1,03	0,78	0,92	1,06	0,49	0,86	0,88
Masculino 15–19 anos	4,47	5,77	6,06	5,87	6,31	6,46	7,48	6,08	7,58	7,52
Feminino 10–14 anos	0,54	0,44	0,76	0,53	0,63	0,68	1,01	0,75	1,14	0,97
Feminino 15–19 anos	3,09	2,79	2,56	2,84	2,88	3,18	3,49	3,08	2,76	3,11
Região Nordeste	2,26	2,43	2,51	2,54	2,62	2,80	3,26	2,61	3,12	3,16
Unidade federativa										
Alagoas	1,34	2,85	2,51	2,84	1,34	2,52	1,69	2,04	1,89	3,48
Bahia	1,18	1,45	1,29	1,56	1,49	2,04	2,34	1,30	2,06	3,13
Ceará	3,16	3,72	4,16	3,90	3,72	3,11	4,15	3,83	4,55	3,80
Maranhão	2,52	2,22	2,78	2,95	4,16	3,29	3,74	2,70	3,17	2,34
Paraíba	2,45	2,62	2,45	1,31	4,08	2,12	3,42	3,42	3,26	1,96
Pernambuco	2,08	2,40	2,00	1,68	2,07	2,72	3,05	2,02	2,37	2,53
Piauí	3,91	3,70	3,89	6,19	2,70	4,43	6,35	5,23	6,66	4,59
Rio Grande do Norte	3,37	1,58	1,78	2,17	1,58	3,56	2,58	3,18	3,41	4,25
Sergipe	2,90	2,62	4,17	2,60	4,15	3,38	3,91	3,15	3,45	4,30

Foi observada uma tendência crescente da taxa de mortalidade por suicídio na região Nordeste. Entre os adolescentes de 10 a 14 anos, foi constatada tendência estacionária para o sexo masculino e crescente para o sexo feminino. Entre aqueles de 15 a 19 anos foi observado o inverso: tendência crescente para os meninos e estacionária para as meninas. Entre os estados da região Nordeste, a maioria mostrou uma tendência estacionária, com exceção da Bahia, que apresentou tendência crescente da taxa (Figura 2).

**Figura 2:** Tendência temporal das taxas de mortalidade por suicídio corrigidas entre adolescentes do Nordeste, segundo sexo, faixa etária e Unidades Federativas, ocorridos de 2011 a 2020.

APC: variação percentual anual.

^a^p < 0,05

Os óbitos por suicídio entre adolescentes representaram uma perda de 11.110 APVP em 2011 e 14.960 em 2020. Os maiores valores ocorreram entre indivíduos de 15 a 19 anos, com destaque para o sexo masculino. Na população total, houve aumento da taxa de APVP de 2011 para 2020. No sexo feminino aumentou de 26,8 para 48,3 entre os adolescentes mais jovens, enquanto no sexo masculino a taxa diminuiu no período estudado. Entre os adolescentes mais velhos, ambos os sexos tiveram aumento da taxa de APVP (Tabela 3).

**Tabela 3:** Anos potenciais de vida perdidos (APVP) devido ao suicídio de adolescentes da região Nordeste do Brasil, segundo sexo e faixa etária, no período de 2011 a 2020.

Faixa etária	Sexo	Óbitos		APVP ^a^	Ponto médio de idade	APVP total		APVP/100.000	
			
		2011	2020	2011/2020		2011	2020	2011	2020
10–14 anos		**36**	**39**	**57,5**	**12,5**	**2.070,0**	**2.242,5**	**39,5**	**46,0**
	Masculino	24	19	57,5	12,5	1.380,0	1.092,5	51,7	43,9
	Feminino	12	20	57,5	12,5	690,0	1.150,0	26,8	48,3
15–19 anos		**166**	**233**	**52,5**	**17,5**	**8.715,0**	**12.232,5**	**173,0**	**243,8**
	Masculino	99	166	52,5	17,5	5.197,5	8.715,0	204,1	343,5
	Feminino	67	67	52,5	17,5	3.517,5	3.517,5	141,3	141,8
Total		**202**	**272**	**55,0**	**15,0**	**11.110,0**	**14.960,0**	**108,1**	**151,3**

^a^ APVP por indivíduo.

## DISCUSSÃO

No período de 2011 a 2020, houve tendência crescente da taxa de mortalidade por suicídio entre adolescentes do Nordeste do Brasil. As maiores taxas foram observadas na faixa etária de 15 a 19 anos, sendo ascendente no sexo masculino e estável no feminino. A maioria dos óbitos ocorreu entre adolescentes de raça/cor parda e com escolaridade baixa. O domicílio foi o principal local de ocorrência e o enforcamento/estrangulamento constituiu o principal método utilizado. Observou-se aumento nos APVP no período estudado.

A tendência crescente das taxas de mortalidade observadas pode estar associada às mudanças instituídas na rede assistencial no período do estudo, como o foco na internação hospitalar e o desmonte dos serviços de saúde de base comunitária^14^. Entre os prejudicados pela nova Política Nacional de Saúde Mental estão os Centros de Atenção Psicossocial (CAPS), que tiveram acentuada redução da implantação de novas unidades^14^, o que implica em desassistência psicológica e psiquiátrica de adolescentes. Tendo em vista que os transtornos mentais são fatores de risco para o suicídio^5,15^, a diminuição do acesso à Rede de Atenção Psicossocial pode potencializar o risco para essa população.

É necessário também considerar a influência do uso das mídias sociais pelos adolescentes da geração atual. Evidências apontam que o tempo excessivo de tela tem relação com o adoecimento mental^16^. A exposição prolongada a mídias sociais tem sido associada a um sono de pior qualidade, *cyberbulling* e maior internalização de problemas, repercutindo em risco de comportamento suicida^16^. Evidências ainda apontam que o aumento dessas taxas pode ter relação com amplas coberturas nos noticiários sobre o suicídio de celebridades, fenômeno conhecido como efeito contágio ou efeito *Werther*, em que o indivíduo se sente encorajado a reproduzir esse comportamento^17^, e isso, somado a características da geração atual, como baixa resiliência e imediatismo, pode influenciar no aumento das taxas de suicídio^2^. Por outro lado, as redes sociais têm também um potencial positivo, por meio das redes de suporte que se formam entre os internautas, evidenciando a necessidade de criar/fortalecer estratégias de prevenção virtuais para alcançar essa população vulnerável^16^.

O predomínio de óbitos por suicídio em adolescentes de raça/cor parda já foi apontado em pesquisa realizada na Bahia^18^. Sabe-se que a maioria da população nordestina se autodeclara parda^19^, e este grupo está entre aqueles classificados como de maior vulnerabilidade social (menores renda, trabalhos informais etc.)^20^ Os índices de suicídio entre adolescentes são positivamente associados às desigualdades socioeconômicas, reforçando a suspeita de que as características de desenvolvimento dessa região possam influenciar no risco de óbito por suicídio^21^.

Neste mesmo sentido, maiores percentuais de suicídios foram observados entre indivíduos com menor escolaridade e do sexo masculino. O baixo rendimento acadêmico e adesão escolar deficiente também já foram identificados como fatores de risco para o suicídio^15^. Muitos adolescentes não conseguem conciliar estudo com trabalho, além de terem menos incentivo dos pais que, em sua maioria, têm baixa escolaridade^20^.

O domicílio constituiu o principal local em que foi cometido o suicídio. Esse dado, além de evidenciar a importância do apoio da família no tocante à prevenção de lesões autoprovocadas, reforça o papel protetor ou de risco que a família pode exercer no comportamento suicida^22^, pois os problemas familiares aumentam o risco de suicídio^23^. Também é possível supor que a maior proporção no domicílio esteja relacionada à facilidade na obtenção dos instrumentos utilizados para cometer o suicídio, bem como à velocidade de concretização do óbito segundo o método^1,18^.

Os hospitais foram identificados como o segundo maior local de ocorrência, sendo mais expressivo entre adolescentes do sexo feminino. Sua escolha de métodos geralmente menos letais pode traduzir o menor desejo de morte e a necessidade de ajuda, uma vez que há mais notificações de tentativa de suicídio e internações por autointoxicação entre elas^3^. No entanto, se a tentativa de reversão do método não é bem-sucedida, os registros de mortalidade em hospitais são elevados^1,24^. Já os meninos, em geral, optam por métodos mais violentos e de alto risco de morte^25^. Isso pode explicar a maior ocorrência de óbitos por esse grupo em domicílio, vias públicas e outros locais que não propiciem assistência médica imediata.

A análise dos meios utilizados fortalece esse entendimento. Embora o enforcamento/estrangulamento tenha sido a principal forma de suicídio em ambos os sexos, assim como o uso de armas de fogo, o percentual foi maior para os meninos. Explicações apontadas para esse comportamento consideram questões culturais que associam a masculinidade à independência e tomada de decisões mais definitivas, além de evitar as repercussões sociais do uso de um método que o possibilite sobreviver e encarar os fatos que o levaram a tentar o suicídio^15^. Em contrapartida, as meninas morreram mais devido a autointoxicação por fármacos e outras substâncias químicas, além de envenenamento de intenção indeterminada, podendo estar associado à maior ocorrência de depressão e possível desejo de sobrevivência, uma vez que são mais predispostas a pedir e aceitar ajuda para enfrentar os seus problemas^15^.

As taxas de mortalidade encontradas neste estudo reforçam essa hipótese, pois os maiores valores predominaram entre os meninos em ambos os grupos etários. Outros estudos realizados no Nordeste e em outras regiões brasileiras também apontaram maior incidência de mortalidade por suicídio entre meninos^7,21,24^. Em relação à faixa etária, a preponderância de óbitos ocorridos entre 15 e 19 anos, em ambos os sexos, pode ser reflexo de constante exposição aos fatores de risco específicos para essa idade, como as dificuldades inerentes à fase de transição para a vida adulta, maior estresse e ansiedade no tocante ao futuro profissional e acadêmico, ao uso excessivo de substâncias psicoativas e a problemas nos relacionamentos familiares e sociais^1,7,15^. Ademais, evidências apontam associação entre o suicídio e adolescentes que não estejam vinculados a um sistema formal de educação, emprego ou treinamento^15^.

A tendência temporal da taxa de mortalidade por suicídio entre adolescentes do Nordeste foi crescente no período estudado, semelhante ao observado em outros estudos^1,7,26^. Na faixa etária de 10 a 14 anos foi observada tendência de crescimento para as meninas e estabilidade para os meninos, diferente do observado em outro estudo ocorrido na região, entre 2001 e 2015, no qual os meninos tiveram tendência em ascensão enquanto as meninas apresentaram estabilidade^7^. A mudança da tendência de mortalidade entre meninas de 10 a 14 anos, em comparação com o estudo anterior, pode ter ocorrido devido à maior exposição delas a conflitos interpares, *bullying* e experiências de abuso emocional, sexual ou físico, os quais já foram apontados como fatores de risco para comportamento suicida, principalmente em meninas jovens^15^. Todavia, no que concerne à faixa etária de 15 a 19 anos, o estudo atual concorda com a evidência de um anterior, que mostrou crescimento da tendência de mortalidade para os meninos e estabilidade para as meninas^7^. Este fato pode estar associado aos meninos serem mais inclinados a ocultar suas fraquezas e adotarem uma conduta mais agressiva na escolha de métodos letais para o suicídio do que as meninas^25,27^.

Em relação às Unidades Federativas do Nordeste, embora a Bahia tenha apresentado uma das menores taxas de mortalidade por suicídio, foi o único estado a apresentar tendência crescente, com variação percentual anual maior do que a observada na sua região. Essa tendência já fora apontada por outros estudos, que analisaram os períodos de 1997 a 2016^1^ e de 2001 a 2015^7^, e pode estar relacionada a aspectos sociodemográficos, uma vez que o estado da Bahia ainda apresenta alta desigualdade de renda, sobretudo ao considerar o aspecto raça/cor, com o rendimento médio domiciliar dos autodeclarados pretos e pardos inferior ao de brancos. Além disso, segundo dados de 2019, a taxa de analfabetismo entre adolescentes negros de 15 anos, na Bahia, foi superior à de brancos^28^ e sabe-se que a escolaridade, junto a outros fatores, pode determinar as oportunidades de emprego e renda^20^.

Os resultados desta pesquisa revelaram que houve diferenças nos APVP por suicídio segundo idade e sexo. Embora a faixa etária mais jovem tenha apresentado aumento da taxa de 2020, em relação a 2011, observou-se que essa elevação foi atribuída a um incremento apenas na taxa do sexo feminino, enquanto a do sexo masculino apresentou diminuição. Isso chama a atenção para uma busca de resolução definitiva cada vez mais precoce dos conflitos internos e externos vivenciados por adolescentes do sexo feminino e os fatores que poderiam estar relacionados a essa precocidade, como violência psicológica e/ou sexual, eventos estressores, uso excessivo das mídias sociais e falta de suporte familiar^3,5,16,26^.

Em relação aos adolescentes mais velhos, em ambos os sexos foi observado aumento da taxa de APVP, sendo mais expressivo no masculino. Isso pode ser atribuído não somente às questões que em geral estão associadas a esse grupo, como maior abstenção escolar, exposição ao álcool e ao desemprego^15^, mas também a um agravamento pelo contexto da pandemia de Covid-19, iniciada no ano de 2020, quando houve um excesso na ocorrência de suicídios na região Nordeste, embora o Brasil tenha apresentado um padrão geral de queda^29^. A pandemia, além das perdas afetivas, reduziu as possibilidades de emprego e de continuação dos estudos, principalmente para aqueles oriundos de escolas públicas e que não tinham recursos adequados à manutenção do ensino remoto. Dados apontam que a redução da ocupação afetou muitos jovens em 2020 e, para essa população, o restabelecimento após crises é ainda mais demorado^8^. Além disso, o contexto pandêmico favoreceu o adoecimento mental, ao produzir um cenário de medo do contágio e a necessidade de isolamento social, o que aumentou os níveis de estresse e ansiedade tanto em indivíduos saudáveis quanto naqueles previamente diagnosticados com transtornos mentais^30^. A soma desses fatores pode ter potencializado a ocorrência do suicídio.

Os achados deste estudo reforçam a relevância do óbito por suicídio entre adolescentes como um problema de saúde pública, ao mesmo tempo em que auxilia na compreensão epidemiológica desse fenômeno. Entre as limitações, destacam-se as restrições das bases de dados que só dispõem as informações por sexo, enquanto a literatura aponta a influência expressiva das características de gênero sobre o risco de suicídio. Além disso, ainda há a grande quantidade de campos ignorados da variável escolaridade e a subnotificação de casos de suicídio, seja devido à dificuldade em determinar intencionalidade do óbito ou por considerar apenas o tipo da lesão, sem ponderar o contexto em que foi produzida. Embora tenham sido incluídas outras categorias da CID-10 na tentativa de minimizar o impacto da subnotificação, cabe ressaltar que outros erros de classificação podem ter ocorrido. O consequente mau preenchimento das declarações de óbito repercute como limitante da implantação e aprimoramento de políticas de prevenção específicas para as populações vulneráveis.

A tendência em ascensão da mortalidade entre adolescentes no Nordeste e no estado da Bahia reforça o peso das características sociodemográficas na determinação da vulnerabilidade que os confere maior risco de suicídio nesses locais. A predominância da mortalidade entre adolescentes de 15 a 19 anos, especialmente do sexo masculino, repercute no aumento dos APVP de uma população em idade produtiva. O aumento da tendência da taxa de mortalidade entre meninas de 10 a 14 anos chama a atenção para a existência precoce de fatores que provocam o adoecimento mental e a crescente busca por medidas drásticas para a resolução de conflitos. Nessa perspectiva, os diferentes perfis de suscetibilidade entre os adolescentes apontam a necessidade de adotar abordagens e formas de prevenção específicas para os fatores que mais predispõem cada grupo ao suicídio, bem como a relevância de explorar o potencial protetor da internet, com o fortalecimento de comunidades de apoio online, mecanismos de busca e oferecimento de ajuda virtual. Além disso, reforça-se a necessidade de ampliação do olhar sobre os fatores de risco, que ultrapassam os aspectos emocionais e sofrem influência de um panorama abrangente, como o contexto sociocultural, o acesso à rede assistencial, à escola e a oportunidades de emprego e renda.

Os resultados deste estudo permitiram identificar o perfil de indivíduos mais acometidos, as formas de execução mais comuns e como as diferenças entre os sexos podem exercer influência sobre a ocorrência do suicídio. Destaca-se a necessidade de intervenções específicas que contribuam para o direcionamento de políticas públicas mais efetivas para promoção de saúde e prevenção do suicídio entre adolescentes.
